# Platelet Satellitism in a Patient with Underlying Infection, Immune Thrombocytopenic Purpura (ITP) and Multiple Sclerosis

**DOI:** 10.3390/diagnostics15111319

**Published:** 2025-05-24

**Authors:** Athanasios Liaskas, Natali El-Gkotmi, Anestis Karapaschalidis, Dimitrios Tzanetakos, Serena Valsami

**Affiliations:** 1 Hematology Laboratory and Blood Bank, Aretaieion Hospital, National and Kapodistrian University of Athens, 11528 Athens, Greece; natelgkotmi@gmail.com (N.E.-G.); anestiskara@hotmail.com (A.K.); 2 Second Department of Neurology, “Attikon” University Hospital, School of Medicine, National and Kapodistrian University of Athens, 12461Athens, Greece; dtzanetakos@med.uoa.gr

**Keywords:** platelet sattelitism, ITP, pseudothrombocytopenia

## Abstract

Platelet satellitism (PS) is an in vitro phenomenon of platelets adhering around white blood cells, especially in blood samples anticoagulated with K_3_EDTA. This, in some cases, can lead to spurious thrombocytopenia, without platelet dysfunction or bleeding events. Diagnosis is made by peripheral blood smear examination. The potential mechanism for PS remains largely unknown; however, it possibly involves the formation of IgG antibodies against the platelet glycoprotein receptor IIb/IIIa (GPIIb/IIIa). PS has been observed in various medical conditions, including infectious, autoimmune, and lymphoproliferative disorders, without an obvious causative relationship. Here, we describe a case of PS in a patient who presented with infection in the setting of underlying Immune Thombocytopenic Purpura and Multiple Sclerosis.

**Figure 1:**
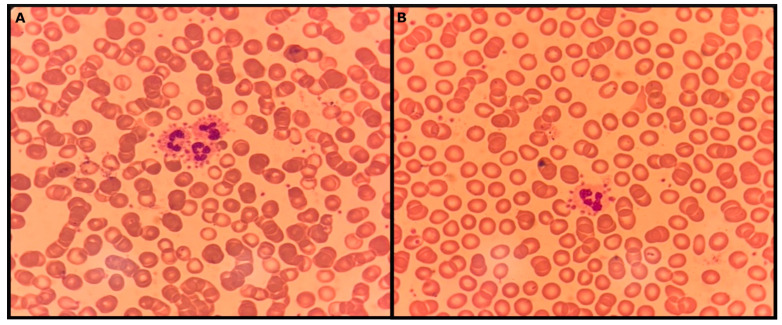
A 44-year-old male patient with relapsing corticosteroid-dependent immune thrombocytopenic purpura (ITP) presented at our department with an acute lower respiratory tract infection. His past medical history was significant for multiple sclerosis (MS) on treatment with alemtuzumab. The complete blood count was unremarkable. The patient’s peripheral blood smear (**A**,**B**) revealed platelet rosetting around neutrophils, a finding known as platelet satellitism (PS). Notably, it was present in every single neutrophil throughout the blood smear. Nevertheless, this did not result in spurious or real thrombocytopenia at that time. Platelet satellitism (PS) was first described in 1963 by Field and McLeod [1], and since then, few cases have been reported in the literature. It is considered an in vitro phenomenon of platelets clustering mainly around neutrophils, especially in blood samples anticoagulated with K_3_EDTA at room temperature [2]. This, in some cases, can lead to spuriously reduced platelet count or “pseudothrombocytopenia”, without platelet dysfunction or bleeding diathesis. The reported frequency of PS is significantly lower than that of EDTA platelet clumping, roughly estimated at 1 per 30,000 CBCs [3]. Potential mechanisms for PS include the formation of IgG antibodies against the platelet glycoprotein receptor IIb/IIIa (GPIIb/IIIa) and the FcγRIII receptor on the surface of neutrophils, leading eventually to the adhesion of the platelets to neutrophils [4,5]. This has been shown in a series of experiments by Bizzaro N et al. by performing inhibition studies with anti-GP Ilb/IIIa and anti-FcγRIII monoclonal antibodies, or by using blood samples from patients with Glanzmann’s thrombasthenia or FcyRIII deficit [4]. Probably in the presence of EDTA, chelation of calcium anions leads to exposure of cryptic epitopes on platelet and neutrophil membranes, enhancing their immune-mediated interaction [6]. Another study proposed a non-immune related mechanism mediated by activation of thrombospondin on the platelet surface (or other α-granule proteins such as P-selectin) [7]. Cases of transient PS in the context of infections, autoimmune and lymphoproliferative disorders, as well as chronic kidney or liver conditions have been described in the literature, implying a possible immunologic mechanism, but without robust explanations regarding the etiology [8,9,10,11,12,13,14,15,16]. Concurrent ITP and PS have been previously described and interestingly one of the already reported cases had ITP and a co-existing neurologic condition (Guillain-Barre syndrome) [17,18,19]. ITP pathophysiology lies in the presence of IgG antiplatelet antibodies against surface glycoproteins mainly GPIIb/IIIa and GPIb/V/IX and less commonly GPIa/IIa, GP IV, and GPVI. Opsonized platelets are destroyed by macrophages predominantly in the spleen through an Fcγ-dependent or complement-mediated mechanism. T-cell alterations are also a key part of the pathophysiology including shifting of T-helper cells towards type 1 (Th1) and type 17 (Th17) helper phenotype, reduction of regulatory T-cell activity, and an increase in cytotoxic T cells [20,21,22,23,24,25]. Currently, there is no robust explanation of the underlying pathophysiology of PS. The role of infection in the context of autoimmunity (MS and ITP) along with immunomodulatory treatment in our patient cannot be overlooked. PS and ITP co-share antiplatelet antibodies with a common target, namely GPIIb/IIIa and this could possibly explain why patients with ITP could have persisting PS. To our knowledge, this is the first case of PS described in the setting of ITP, MS, and infection. PS is exclusively diagnosed by peripheral blood smear and represents an incidental in vitro phenomenon with a benign clinical course. Prompt diagnosis of PS retains its clinical significance as other causes of spurious thrombocytopenia could be excluded, avoiding unnecessary diagnostic and therapeutic interventions.

## Data Availability

Not applicable.
